# Patients' and Observers' Perceptions of Involvement Differ. Validation Study on Inter-Relating Measures for Shared Decision Making

**DOI:** 10.1371/journal.pone.0026255

**Published:** 2011-10-17

**Authors:** Jürgen Kasper, Christoph Heesen, Sascha Köpke, Gary Fulcher, Friedemann Geiger

**Affiliations:** 1 Institute of Neuroimmunology and Clinical MS Research (INiMS), University Medical Center Hamburg, Hamburg, Germany; 2 Unit of Health Sciences and Education, MIN-Faculty, University of Hamburg, Hamburg, Germany; 3 MIN-Faculty, Institute of Health Sciences and Education, University of Hamburg, Hamburg, Germany; 4 MS Australia NSW, Lidcombe, Australia; 5 Tumor Center, University Medical Center Schleswig-Holstein, Kiel, Germany; 6 Department of Pediatrics, University Medical Center Schleswig-Holstein, Kiel, Germany; Innsbruck Medical University, Austria

## Abstract

**Objective:**

Patient involvement into medical decisions as conceived in the shared decision making method (SDM) is essential in evidence based medicine. However, it is not conclusively evident how best to define, realize and evaluate involvement to enable patients making informed choices. We aimed at investigating the ability of four measures to indicate patient involvement. While use and reporting of these instruments might imply wide overlap regarding the addressed constructs this assumption seems questionable with respect to the diversity of the perspectives from which the assessments are administered.

**Methods:**

The study investigated a nested cohort (N = 79) of a randomized trial evaluating a patient decision aid on immunotherapy for multiple sclerosis. Convergent validities were calculated between observer ratings of videotaped physician-patient consultations (OPTION) and patients' perceptions of the communication (Shared Decision Making Questionnaire, Control Preference Scale & Decisional Conflict Scale).

**Results:**

OPTION reliability was high to excellent. Communication performance was low according to OPTION and high according to the three patient administered measures. No correlations were found between observer and patient judges, neither for means nor for single items. Patient report measures showed some moderate correlations.

**Conclusion:**

Existing SDM measures do not refer to a single construct. A gold standard is missing to decide whether any of these measures has the potential to indicate patient involvement.

**Practice Implications:**

Pronounced heterogeneity of the underpinning constructs implies difficulties regarding the interpretation of existing evidence on the efficacy of SDM. Consideration of communication theory and basic definitions of SDM would recommend an inter-subjective focus of measurement.

**Trial Registration:**

Controlled-Trials.com ISRCTN25267500.

## Introduction

The aim of evidence based medicine (EBM) is to provide the means by which current best evidence from research can be applied to medical decision making [1]. Since evidence alone does not make decisions [2], such means are not exhausted by generation, synthesis and appraisal of research evidence. They rather imply providing evidence to patients in a way that allows them to make an informed choice [2]. The latter has been conceived as the ‘shared decision making' method (SDM), a communication strategy to involve patients into the process of making their medical decisions.

Following this concept, patient involvement implies a two way exchange of information between doctor and patient where options are made explicit, appraisal of current best evidence is negotiated, and patient desires are elicited [3]. This style of communication contrasts the traditional benevolent paternalism where patients are assigned to a passive role in the decision making process [3]. Emphasizing its relevance for the quality of healthcare, SDM can be seen as a key method in realizing the underpinning goals of EBM. Apart from ethical guidelines [4] and patients' pronounced role preferences for more participation in decision making [5], this view is supported by efficacy studies. SDM or interventions intending to facilitate SDM has been shown to improve decision quality by enhancing knowledge, patient satisfaction with the decision making process and realistic expectations, or by decreasing fears and decisional conflict [6].

Other studies evaluating SDM interventions have found no effects on communication, patient satisfaction or on health status [6]–[9]. Theory is lacking to predict conditions under which SDM can yield desired effects [10]. Apart from this, evidence on efficacy of SDM can just be considered meaningful to the extent to which the communication measurement is valid. However, too little attention has been given to the issue of SDM measurement [11]–[14].

While most instruments address associated dimensions such as patients' decision making needs, decision support, satisfaction or the feeling of being informed, few instruments address aspects of the communicative process. These vary with regard to their level of validation and to the perspective from which SDM is assessed: the observer's, the physician's or the patient's perspective. Most feasible to administer, a few patient questionnaires exist to assess perceived quality of the decision making process in terms of either the feeling of being informed, supported and taken serious with one's individual preferences (Shared Decision Making Questionnaire, SDMQ, Perceived Involvement in Care Scale, PICS and Decision Conflict Scale DCS [11]), or in terms of the social role model between patient and physician in the consultation (Control Preference Scale CPS [11]). Another promising method is an observation based rating scale (OPTION = Observing Patient Involvement [15]), providing criteria to appraise the physician's behavioural efforts to involve the patient. OPTION has already been used in many countries and settings [16]–[19].

All these measurements approach patient involvement using a unilateral perspective as a proxy for SDM. Although proofs of validity for these instruments in some regard have been published [14], e.g. showing the OPTION scale sensitive to physicians' communication behaviour [15], their validity with regard to patient involvement as an interpersonal process has not yet been investigated. It is, however, evident from communication theory that a two way exchange of information and the shared appraisal and negotiation of a decision making process is a dynamic interpersonal process not operable from any unilateral perspective [20].

Our study therefore aimed to examine the OPTION scale's ability to indicate patient involvement in physician patient consultations as perceived from the patient perspective. Assuming that (as a proof of validity) SDM measures administered from varying viewpoints should correspond, we applied OPTION and three measures assessing SDM from the patients' perspectives to the same pool of consultations. By yielding empirical evidence on the degree of interrelatedness of commonly used approaches to SDM this study also contributes to the debate on conceptual issues and underpinning assumptions which are reflected by these measurement approaches [20].

## Methods

### Ethics statement

The study protocol was approved by the ethics committee of the Hamburg Chamber of Physicians, and all participants gave written informed consent for record, analyses and publication of their data collected within this study.

### Umbrella study

**Figure 1:**
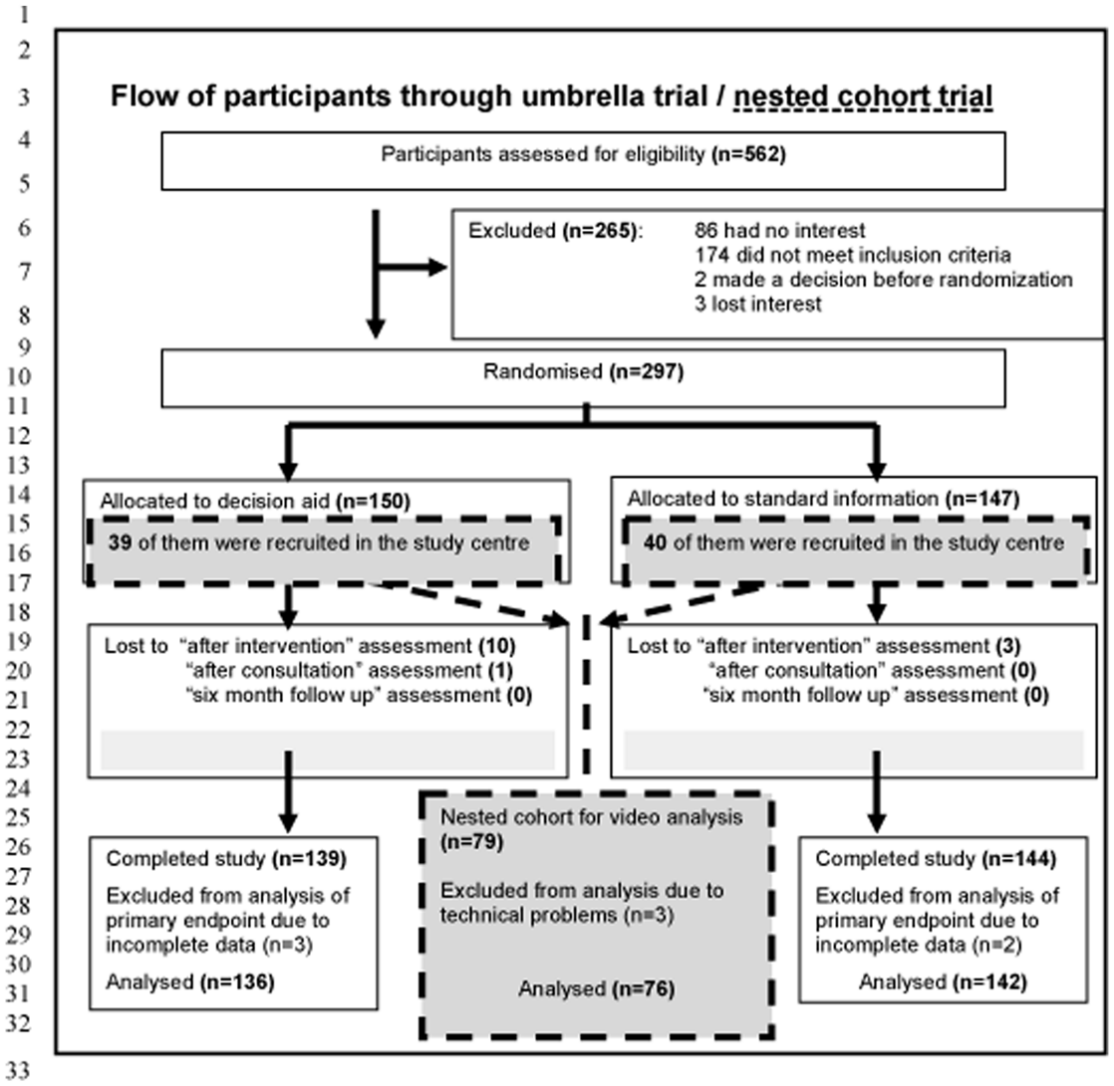
Flow of participants through umbrella trial and nested cohort trial.

We studied a nested cohort of a randomized controlled trial (RCT) [8] evaluating the effectiveness of a patient decision aid developed to support people with multiple sclerosis (MS) in deciding on immunotherapy (Figure 1) referred to as umbrella study. Overall, 297 patients were included in this umbrella study, recruited mainly through press advertisements published throughout Germany, but also directly at the main study centre in Hamburg. To obtain deeper insights into the communication we asked all patients recruited at the Hamburg study centre to agree to video recording of their consultations with the physician. These records served as sample of communication behaviour for the present study. The development of the decision aid and the results of its effects have been reported elsewhere [8], [21].

### Content of the decision and context

Accompanied by the physician on duty, participants were either considering whether to start immunotherapy or reconsidering their current immunotherapy. Depending on the course and stage of the disease, different kinds of immunotherapy are available. Accordingly, the number of options varied from case to case as well as the probabilities of benefit. Neither further disease course nor chance of benefit can be predicted in an individual case. Moreover, long-time effectiveness of immunotherapy is a matter of debate [22]. Since patients have to weigh up uncertain benefit and considerable side effects, this decision is highly appropriate for a shared decision making process [23]. As an inclusion criterion of the umbrella trial, all patients had an actual decision to make and, as a consequence of the design of the umbrella trial, the decision was made within these consultations.

### Measurement

The umbrella study collected data at 4 measurement points: baseline (T0), after intervention (T1), directly after consultation (T2), and about 5 months after consultation (T3) [8]. Beyond the demographic and disease related data, treatment choice and different scores evaluating the communication were recorded. The nested cohort study is based on four different judgements of patient involvement: one administered by objective observers based on video documents; three administered by the patients, two of these directly after the consultation (T2); and one six months after randomisation (T3).

### OPTION scale

The OPTION scale [16] is typically administered by an observer watching the physician-patient conversation, and then scoring the physician's offers to involve the patient on a five point Likert scale (0 = ‘not observed’; 4 = ‘executed to a high standard’) (Table 1). In a chronological order, 12 items cover the decision making process beginning at a precise statement referring to the subject of the particular decision and ending in the decision itself and the follow up statements [16]. The analyses conducted in this study were based on video documents (T2). All consultations were analysed by JK, who had been trained to use the coding manual by the scale's principal author. Videos were analysed in random order, and the rater was blind to any other study data of the patients. Rater training was simultaneously given to an advanced student (GB). This involved each judgement being explicitly deduced and comprehensively explained on the basis of the manual for trainees. This proceeding was intended to maximise reliability of the observer data. Independently, the trainee recorded her own ratings referring to each video. In cases of doubt or disagreement, the video was analysed again. In addition, a record was made of problems relating to the applicability of the rating instrument, such as obvious limitations of distinctiveness or exhaustivity.

**Table 1:** Observed communication competences and reliability.

OPTION item	Mean(SD)	InterRR	IntraRR
1) The clinician *draws attention to* an identified problem as one that requires a decision making process.	1.2(1.2)	.92	.98
2) The clinician *states* that there is more than one way to deal with the identified problem (‘equipoise’).	0.8(1.0)	.83	.93
3) The clinician *assesses* the patient's preferred approach to receiving information to assist decision making (e.g. discussion, reading printed material, assessing graphical data, using videotapes or other media).	0.05(0.3)	1	1
4) The clinician *lists* ‘options’, which can include the choice of ‘no action’.	0.7(0.9)	.87	1
5) The clinician *explains* the pros and cons of options to the patient (taking ‘no action’ is an option).	1.5(0.9)	.87	.90
6) The clinician explores the patient's *expectations* (or ideas) about how the problem(s) are to be managed.	2.2(0.8)	.73	.91
7) The clinician explores the patient's *concerns* (fears) about how problem(s) are to be managed.	1.8(1.9)	.76	.78
8) The clinician checks that the patient has *understood* the information.	0.1(0.5)	1	1
9) The clinician offers the patient explicit *opportunities* to ask questions during the decision making process.	1.4(0.7)	.99	.90
10) The clinician elicits the patient's *preferred level of involvement* in decision-making.	0.8(0.6)	.50	.89
11) The clinician indicates the need for a *decision making* (or *deferring*) stage.	1.4(1.1)	.83	1
12) The clinician indicates the need to review the decision (or *deferment)*.	2.4(1.4)	.67	.95
Mean	1.2(0.4)	.83	.94

Item range 0–4: 0 = skill not observed, 4 = skill executed to a high standard; InterRR = inter-rater reliability, based on 26 consultations IntraRR  = intra-rater reliability, based on 15 consultations (Correlation coefficients are based on Spearman).

### Shared Decision Making Questionnaire (SDM-Q)

The SDM-Q was used to assess patients' view on the consultations. The questionnaire follows the same taxonomy of decision making steps as the OPTION scale and was developed to show the extent to which patients felt they were involved in the process. In its revised form, SDM-Q has 11 items scoring from 0 to 3 on a 4 point Likert scale [24]–[25]. Patients received the questionnaire by mail for self-administration after the consultation (T2).

### Control Preference Scale (CPS)

The CPS [26] presents subjects with a choice of five alternative decisional roles and requires them to identify the one that best describes their preferred position. In the present study CPS_post_ was used, which is supposed to evaluate the role position after a consultation. CPS_post_ was sent to patients as a multiple choice questionnaire and assessed at T2 during a telephone interview. According to Degner [26], the 5 descriptions of social role distributions in the physician-patient-interaction were: 1: “I made my decision alone”, 2: “I made my decision alone considering what my doctor said”, 3: “I shared the decision with my doctor”, 4: “My doctor decided considering my preferences”, 5: “My doctor made the decision”. The CPS_post_ also included the answer “the decision was deferred”.

### Decisional Conflict Scale (DCS)

The DCS [27] was presented at T3 in a form slightly adapted to the specific decision by exchange of abstract terms by terms referring to the particular decision on immunotherapy. The 16 items scoring from 0 to 4 on a 5 point Likert scale includes five subscales: certainty (3 items), information (3 items), values (3), support (3), and quality of the decision (4 items). The latter and the scale's mean score can be understood as a global rating of the degree the patient feels comfortable with the decision. Moreover, since most items address issues evaluating the process rather than the result of making a decision and these items cover the characters of an ideal SDM, many authors used the DCS as a measure for quality of the decision making process in terms of SDM.

### Hypotheses

Considering the raters' intensive training and previous experience and the homogeneity of the sample, we expected to find high levels of inter- and intra-rater reliability when applying the OPTION scale. The four instruments included in this study are all approaching patient involvement via a proxy by accessing a single aspect theoretically associated with the construct, such as the physician's skills (OPTION), the patient's perception or evaluation of the decision (DCS, SDM-Q), and the realized role model (CPS_post_). While being aware of the widespread use of these instruments to measure SDM, we attributed this habit to the lack of appropriate measures of the construct rather than to their factual ability to capture the same construct. Therefore, and based on theoretical considerations [20], we expected to find OPTION, SDM-Q, CPS_post_, and DCS at most moderately interrelated. We expected, however, higher correlations between single item pairs of OPTION and SDM-Q addressing identical content.

### Methods of analysis

After training with 50 consultations, inter-rater-reliability (IRR) was calculated based on the remaining subsample of videos using Spearman correlation coefficients. Additionally, 15 randomly selected videos were rated again by one of the raters (JK) after one year to ascertain intra-rater-reliability. CPS responses were lumped for analysis reducing the number of options from five to three [8]. OPTION scores were transformed to a 0–100 scale as recommended [16]. To explore the relationships between SDM measures mean scores from OPTION, SDM-Q and DCS were correlated pair-wise (Spearman). In our nested cohort, global correlations (in the pooled sample) could potentially derive from locally uncorrelated data (for each physician and for group allocation) and vice versa. Therefore, local correlations within each physician contributing enough consultations and for each group were calculated. Three patient groups defined by CPS response were compared regarding potentially varying communication indices (OPTION, DCS and SDM-Q) using Kruskal-Wallis test. Single pairs of OPTION and SDM-Q items with equal or similar content were identified and Spearman correlations were calculated within these pairs.

## Results

During the umbrella trial, 79 (of 297) participants were recruited by the Hamburg study centre. All participants consented to video recording. 76 of the 79 videos were useable (three could not be analysed for technical reasons). Four physicians were involved in consultations with this subsample. The subsample was comparable to the total sample with regard to demographic and disease related variables [8]. Inter-rater reliability for the OPTION scale was high (rho = .83) and intra-rater-reliability was very high (rho = .94) calculated based on 26 videos (Table 1). According to OPTION, the physicians' performance was on the level of “making attempts to involve the patient” (mean = 30) (Table 1). The physicians showed some differences in their mean scores (27.5 to 37.5) which were significant (p = .009) due to low intra-group variance (SD = 10). In contrast, patients' reports of perceived involvement were quite positive (SDM-Q mean = 2.4, SD = .56). These values did not differ from the values obtained from the total sample.

**Figure 2:**
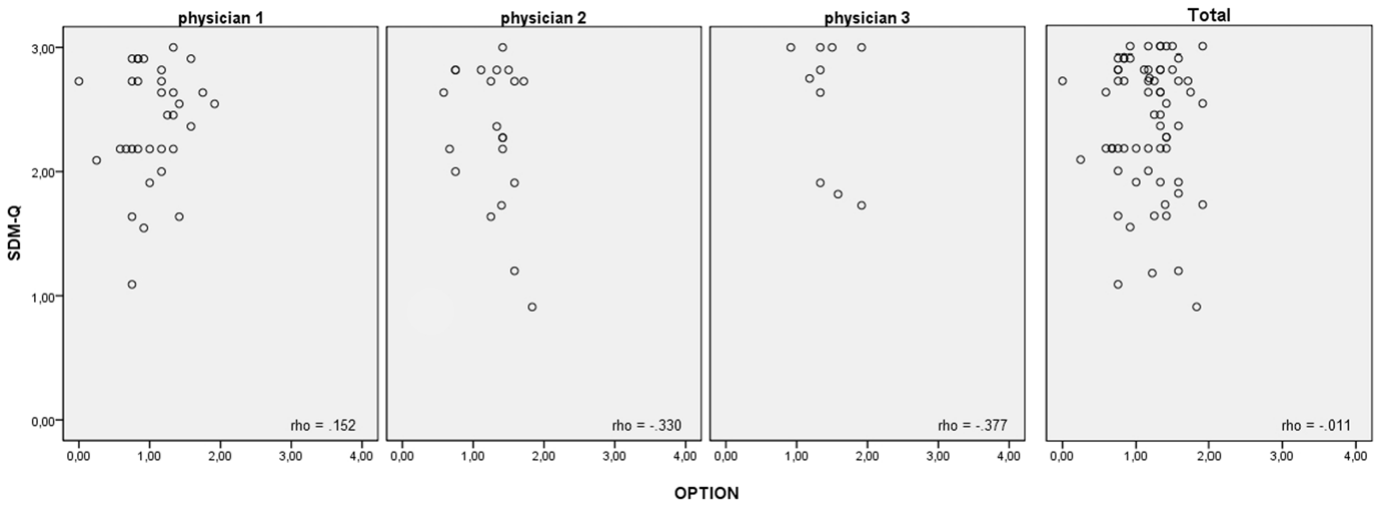
Relationship of OPTION and SDM-Q. Each point represents one consultation. Data are given separately for physicians 1 to 3 and for the whole sample (physician 1: n = 36, physician 2: n = 23, physician 3: n = 14, physician 4 n = 3). Correlations are indicated by Spearman's rho.

**Figure 3:**
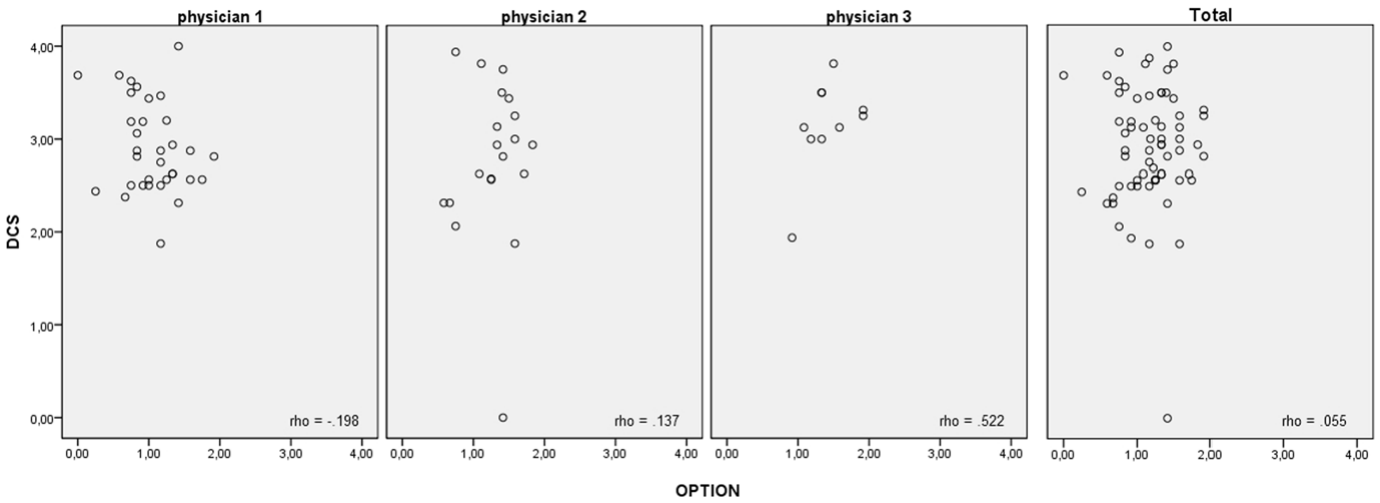
Relationship of OPTION and DCS. Each point represents one consultation. Data are given separately for physicians 1 to 3 and for the whole sample (physician 1: n = 36, physician 2: n = 23, physician 3: n = 14, physician 4 n = 3). Correlations are indicated by Spearman's rho.

The investigation of the relationships between SDM measures revealed extensive incongruence between the four instruments (Figure 2, 3). Virtually no correlation was found between OPTION mean score and SDM-Q mean score (rho = −.01,p = .93), between OPTION mean score, DCS mean score (rho = .05,p = .66), and the mean scores of the five DCS subscales (certainty, information, validation, support, quality: rho = .00 to .13,p = .99 to .28). Moreover, Kruskal-Wallis test revealt no relations between the self-reported role position within the dyad (CPS_post_) and SDM as measured by OPTION (p = .87) or decisional conflict (p = .23). Accordingly, correlations within each physician and within each condition of the umbrella study (decision aid vs. standard information) were comparably low (Figure 2). However, perception of a more autonomous role (CPS_post_) was associated with more involvement as reported in SDM-Q (Kruskal-Wallis test, p<.001 in the total sample). Accordingly, SDM-Q was moderately correlated with DCS (rho = .38,p<.001). Even four (SDM-Q & OPTION) item pairs with identical content yielded uncorrelated data (Table 2). This also holds for analysis of physician-based clusters and within each condition.

**Table 2:** Pair-wise item level correlations of observers' and patients' views.

SDM issue	SDM-Q item number	OPTION item number	Spearman's rho	p-value
opportunity to ask questions	2	9	−.04	.77
consideration of pros and cons	6	5	−.06	.62
follow up arrangement	10 / 11	12	.06 / .04	.63 / .79

Item pairs with identical semantic were selected.

## Discussion

### Principal findings

This paper presents one of the few studies applying multiple SDM measurement techniques to a specific consultation [19], [28]–[31]. It thus provides an opportunity to explore the degree to which their underpinning constructs are empirically congruent. With regard to the clarity of our results, it can even be challenged whether any of these measures has the ability to validly assess SDM.

Our study found that observations of patient involvement in decision making processes about immunotherapy as judged from physicians' behaviour using OPTION were completely unrelated to the patients' reports of being involved (SDM-Q), their level of decision autonomy (CPS_post_), and their evaluation of the decision quality (DCS). The correlations between instruments focussing on the patients' perception of the communication were in part significant but even these only moderately (CPS_post_ / SDM-Q and DCS/ SDM-Q).

### Limitations

These results were yielded based on data drawn from a convenient sample in one of the study centres of the umbrella trial. Due to this strategy and the limited number of involved physicians all belonging to the same unit the communication material might not be representative for other medical contexts, patient populations or patient-physician dyads. As far we were able to examine this, our results are robust with regard to potential biases caused by different physicians or by properties of the instruments used, such as reliability, variability or the considerable ceiling effect. All correlations between CPS, DCS and SDM-Q remained unchanged when calculated for the total sample of the umbrella study. It may be argued that adjusting the alpha level due to multiple testing of correlations would have been appropriate to avoid identification of false positive correlations. However, as in this study there were hardly any significant correlations, adjustment would not have changed our main conclusions. As shown by others [16]–[19], the OPTION scale turned out to be applicable with high levels of reliability. During our work with the instrument, we felt however increasingly critical of its ability to capture the involvement taking place. The conceptual limitation to assessment of physicians' behaviour under some conditions leads to some noteworthy paradoxes. Doctors allowing patients' to involve themselves actively by initiating SDM behaviour are poorly evaluated for omitting the latter. Apart from this, we found the OPTION scale's selection of items incomplete and laying higher emphasis on the doctor's compared to the patient's parts. For instance, apart from the item assessing the physician's efforts to reassure the patients understanding (Item 8), we missed a corresponding item assessing his/her understanding of the patient's point of view. Moreover, OPTION does not include disclosure of the source of recommendations and information (e.g. own experience, scientific evidence, own preferences e.g. due to a conflict of interest), which is an important quality marker of evidence based risk communication [32].

It can be challenged that our sample might not be representative for other decisional settings. The type of decision, however, with regard to pronounced uncertainty within a chronic condition seems paradigmatic for SDM and choices in health care in general. Moreover, SDM can be meaningfully applied whenever a decision between more than one option is to be made. We see no reason why SDM measurements should work dependent on the specific context or course of a decision. Results may not be generalizable to dyads performing higher levels of patient involvement than seen in this study, which, however, were quite comparable to those reported from other studies [15], [17]–[18]. Since there was nevertheless enough variance in the OPTION scores to reach excellent reliability indices, we would not call this poor performance a floor effect. The delay between the record of DCS and the other three measures limits their comparability. As DCS is constructed rather as a measure of decision quality, it can be questioned from a theoretical point of view to which degree the DCS covers the same construct as OPTION, SDM-Q and CPS_post_. However, at least three scales, ‘feeling sufficiently informed’, ‘having had opportunity to consider values and preferences’, and ‘feeling supported by the doctor’ meet the core issues addressed by the other scales. Building convergent validities therefore is well founded. Empirically, DCS was as little related to the other patient administered measures as they were to each other.

### Results in context

Other studies support our findings of inconsistency within SDM as measured from different perspectives. In a study of 212 doctor–patient consultations in general. practice, there was only moderate agreement between patient perceptions of their level of involvement in decision making and the objective ratings using the Evidence Based Patient Choice Instrument [33]. In a validation study of the Rochester Participatory Decision Making Scale, objective behaviour of general practitioners was only weakly correlated with simulated patients' views on “health climate” and “physician trust” and largely uncorrelated with “finding common ground” [28]. From the present study we cannot report data about the congruency of physicians' and observers' perspectives or of physicians' and patients' perspectives. However, it is known that patients and physicians often have disparate experiences regarding their encounters [29], [31], [34]–[37]. It has even been shown that the parties' perceptions of the physicians' efforts to involve patients can be diametrically opposed to one another. Paradoxically, sometimes the fewer options offered to the patients, the more they feel involved in the decision making process [30]. Patients lose trust in physicians verbally expressing uncertainty [38]–[39]. The phenomenon of discordance appears even within the same perspective: In accordance with the present study discrepant assessments of the same decision-making situation by the same patients using different measures have already been shown by others [25], [40]–[41].

Our results touch some basic questions of SDM research with far-reaching implications regarding methods and concept:

### Implications

#### The relevance of the measurement perspective

The results show that existing SDM instruments are not measuring the same construct. This finding is disconcerting with regard to assumptions that are apparently commonly made when these measures are used, or the results they generate are reported. In particular, at least SDMQ and OPTION explicitly refer to the same construct, that is the “extent of patient involvement in the process of decision making” [11], [15]. With respect to face validity and by partly using similar items, DCS and CPS_post_ seem to address a similar definition of the SDM core-construct. This raises the question as to who has the valid perspective: The observer, who is independent of the event and therefore should be a reliable source?; The physician, who is more or less biased by interfering constructs and interests?; or the patient, who is-after all-the main protagonist, but is nevertheless unaware of the criteria of evidence-based patient information and shared decision making?

### Considerations regarding construct and concept

Apart from the problem of the valid perspective, our result may reflect conceptual deficits in SDM. Systematic consideration of the existing definitions of SDM reveals widespread use of the term although a clear and operational basic definition has not yet been agreed [42]–[43]. There is also evidence supporting suggestions that patients do not want to be involved along the academic taxonomies. Patients' conceptualisation of patient involvement contrasts the emphasis within the dominant scientific discourse about patient involvement [44]. As considered by patients in addition to the more readily observable aspects of action and information exchange, involvement does have a relational dimension perceived by patients via more subtle qualities such as the tone or manner of doctors' communication mediating caring, concern, respect and compassion [9], [44]. It has also been shown that patients tend to understand the concept of participation in the process of making a decision in terms of being involved with the doctor in a relational sense [45]. However, these patient-sided concepts of involvement can doubtlessly be properties of a paternalistic communication style as well. Additionally, skills to foster individual decisions made by autonomous patients may, on the other hand, even be seen as associated with more social distance within the dyad [46].

Against this background, our results could highlight some unwanted side effects of SDM communication techniques. Incorporation of these conceptual considerations even seems to complicate attempts to agree on a SDM core construct and to assess the extent or quality of patient involvement. We therefore want to draw the readers' attention to a hitherto mostly neglected character of SDM.

### Intersubjectivity

As interaction is more than just two person's actions, involvement is an interpersonal event to be considered on an intersubjective level. A key role of intersubjectivity in SDM as claimed by some authors [13], [47]–[48] is in line with its basic definition as a “two-way exchange of information” [3] and would imply that, finally, no single perspective could ever indicate SDM. Therefore, lacking correspondence between unilateral SDM measures might just result from the fact that taking each of the measures separately, none of them is touching the intersubjective quality of the construct. Moreover, phenomena of disagreement in the appraisal of a communication, as discovered by this and other studies, might, rather than an error of measurement, be highly critical for the quality of communication in terms of SDM. Instead of trying to avoid disagreement, this assumption would imply a need to address interpersonal disagreement by measurements. Therefore, attention to both participants' as well as observers' perspectives is needed to allow for analyses on a dyadic or triadic data level [20], [49]–[51].

### Conclusion

The study casts a critical light on current SDM research by indicating substantial limitations regarding the validity of existing SDM measures with substantial implications for the interpretation of SDM efficacy studies. Deeper analysis of the methods of measurement also revealed weaknesses in the definitions of SDM. However, inconsistencies between SDM measures and potential interference of subjective and theoretical concepts of communication quality can inform a better understanding of the intersubjective core of the SDM concept. This concept can be better enunciated using more sophisticated strategies to investigate communication, such as dyadic analysis [49]–[51].
